# Factors influencing the effectiveness of multisource feedback in improving the professional practice of medical doctors: a systematic review

**DOI:** 10.1186/1472-6920-14-76

**Published:** 2014-04-11

**Authors:** Julie Ferguson, Judy Wakeling, Paul Bowie

**Affiliations:** 1 NHS Education for Scotland, 3rd Floor, 2 Central Quay, 89 Hydepark Street, Glasgow G3 8BW, Scotland; 2 Queen Margaret University, Edinburgh EH21 6UU, Scotland

**Keywords:** Systematic review, Multisource Feedback, Physicians, Assessment, Revalidation

## Abstract

**Background:**

Multisource feedback (MSF) is currently being introduced in the UK as part of a cycle of performance review for doctors. However, although it is suggested that the provision of feedback can lead to a positive change in performance and learning for medical professionals, the evidence supporting these assumptions is unclear. The aim of this review, therefore, was to identify the key factors that influence the effectiveness of multisource feedback in improving the professional practice of medical doctors.

**Method:**

Relevant electronic bibliographic databases were searched for studies that aimed to assess the impact of MSF on professional practice. Two reviewers independently selected and quality assessed the studies and abstracted data regarding study design, setting, MSF instrument, behaviour changes identified and influencing factors using a standard data extraction form.

**Results:**

A total of 16 studies met the inclusion criteria and quality assessment criteria. While seven studies reported only a general change in professional practice, a further seven studies identified specific changes in behaviour. The main professional behaviours that were found to be influenced by the feedback were communication, both with colleagues and patients and an improvement in clinical competence/skills. The main factors found to influence the acceptance and use of MSF were the format of the feedback, specifically in terms of whether it was facilitated, or if narrative comments were included in the review, and if the feedback was from sources that the physician believed to be knowledgeable and credible.

**Conclusions:**

While there is limited evidence suggesting that MSF can influence professional performance, the quality of this evidence is variable. Further research is necessary to establish how this type of feedback actually influences behaviours and what factors have greatest influence.

## Background

Medical practitioners are now professionally accountable via periodic revalidation for the standard of patient services they provide [1]. The assessment of professional performance is one way to demonstrate fitness to practise medicine and provide evidence of accountability. Medical regulators and educational bodies are endeavouring to identify reliable and robust methods to routinely assess the competence of trainee and qualified doctors to contribute supporting evidence of ongoing performance [2,3]. However, while there is agreement on the need for regular performance assessment of doctors, how best to do this is unclear. Assessment methods involving simulated patients, video observation, audits of clinical records, critical incident analysis and multisource feedback are all suggested, but these approaches involve subjective judgements and lack adequate psychometric evaluation [2,4-7]. An earlier systematic review of a diverse range of assessment methods highlighted the lack of rigour, effectiveness and overall utility of most approaches [8]. However, in terms of costs and time considerations, feedback from colleagues (also known as multisource feedback) was deemed to be the most appropriate and practical method. Moreover, whilst MSF may be seen as a collection of subjective assessments, studies of assessment methods have concluded that subjective methods can be as reliable as objective methods provided the sample size of assessors is adequate [9,10].

As such, multisource feedback (MSF) is being introduced by the UK medical regulator, the General Medical Council (GMC), [11] as part of a cycle of performance review for all medical doctors. The GMC asserts that this feedback needs to be from more than a single source (e.g. self, medical colleagues and non-medical co-workers) and cover the whole of the individual’s professional practice [11].

MSF is a questionnaire-based process, which allows for the feedback to be collected in a systematic way [11]. The intent of the feedback is to guide behaviour change and improve performance [12]. The participant usually receives a copy of their own data (presented as aggregate scores for each element of perceived professional performance being assessed by colleagues) along with comparison data for their group. Graphical as well as numerical data may be provided to the participant. Obtaining more than one person’s view of colleague performance means that a “more complete and accurate assessment of the employees’ competencies” [12] is generated. Many MSF tools also require participants to self-assess, by completing the questionnaire themselves as a means of comparing self and others’ perceptions of professional performance [13].

Although it is suggested that the provision of feedback can lead to learning and a positive change in performance for medical professionals, the evidence supporting these assumptions is unclear [14]. Even with studies which have reported performance improvement over time, it is uncertain if this is attributable to the MSF process or some other mechanism. For example, a prospective longitudinal study which collected MSF data from 250 family physicians on two occasions, five years apart, did find improvements in behaviour, but due to the large gap in time between the two assessments, whether this improvement was attributable to behaviour change as a result of MSF participation was not clear [15].

Therefore, while participation in MSF is now compulsory for all medical doctors, an exploration of the factors that influence the effectiveness of MSF is a timely one, as it would ensure that MSF is delivered in a way that facilitates acceptance and behaviour change. The purpose of this literature review, therefore, was to identify the key factors that influence the effectiveness of multisource feedback in improving the professional practice of medical doctors. To achieve this we conducted a systematic review of the published evidence to address the following questions:

• What impact does MSF have on the professional practice of medical doctors?

• What are the main factors of MSF that influence its impact on the professional practice of medical doctors?

## Methods

This study was conducted and reported according to PRISMA guidelines [16].

### Search Strategy

The following electronic bibliographic databases were searched from inception to November 2012 for primary studies: MEDLINE; EMBASE; CINAHL; PsycINFO; Psychology and Behavioral Sciences Collection; Cochrane CENTRAL Database; Applied Social Sciences Index and Abstracts (ASSIA). The search strategies employed included the following terms: peer review, colleague feedback, multisource feedback, psychological feedback, 360 degree feedback and health professional. These words were searched as a combination of key words, and MeSH subject heading terms. The full Medline search strategy is in below:

**Search strategy for MEDLINE** (**searched from inception** – **November 2012**)

1. Exp Peer Review, Health Care/

2. (feedback adj3 (review$ or colleague or “360” or peer)).mp

3. (multisource or multi-source or “multisource) adj3 feedback.

4. ((colleague or peer) adj3 assessment).mp

5. “360 degree evaluation”.mp

6. Feedback, Psychological/

7. 1 or 2 or 3 or 4 or 5 or 6

8. Exp Health Personnel/

9. 7 and 8

To augment the database searches the reference lists of previous literature reviews and of all retrieved eligible studies were reviewed. The journals, *Medical Education*, *Academic Medicine*, *British Medical Journal*, *Education for Primary Care*, *Journal of the American Medical Association* and *Journal of Continuing Education in the Health Professions* were also hand searched from November 2002-November 2012.

### Inclusion and exclusion criteria

Published empirical studies with a focus on the development and application of MSF methods by all healthcare professional groups were initially to be included (except literature reviews, as the primary studies reviewed would already have been included). However, a preliminary scoping review identified potentially eligible studies which involved medical professionals only. The focus of this review, therefore, is on published work of relevance to the medical professions.

To evaluate outcomes, Barr’s (2000) adaptation of Kirkpatrick’s four level evaluation model [17] was applied to all study findings (Table 1). Our focus was on capturing changes in professional behaviours, so levels 3a (self-reported change in behaviour) and 3b (measured change in performance) were of direct relevance. Identified studies, therefore, had to report evidence of the assessment of at least one of these outcomes in order to be included. Studies concerned with psychometric development and validation and others that only measured knowledge or performance in a test situation, or behaviour not unique to the clinical environment (e.g. teaching or academic research) were excluded.

**Table 1:** **Barr**’**s adaptation of Kirkpatrick**’**s four level evaluation model**

**Level**	**Description**
**Level 1: Learners’ reaction**	Relate to participants’ views of their learning experience and satisfaction with the programme.
**Level 2: Learning outcomes**	
** Level 2a: Modification of attitudes/perceptions**	Changes in reciprocal attitudes or perceptions between participant groups, towards patients/clients and their condition, circumstances, care and treatment.
** Level 2b: Acquisition of knowledge/skills**	Acquisition of concepts, procedures and principles of interprofessional collaboration or the acquisition of thinking/problem-solving, psychomotor and social skills linked to collaboration
**Level 3: Change in behaviour**	Behavioural change transferred from the learning environment to the workplace prompted by modifications in attitudes or perceptions, or the application of newly acquired knowledge/skills in practice. Overeem et al. (2010) identify that this level can be further separated into:
** Level 3a: Self reported change in behaviour**	
** Level 3b: Measured change in performance**	
**Level 4: Patient/Organisational outcomes**	
** Level 4a: Change in organisational practice**	This relates to wider changes in the organisation/delivery of care, attributable to an education programme.
** Level 4b: Benefits to patients/clients**	Covers any improvements in the health and well being of patients/clients as a direct result of an education programme.

Studies identified through the literature searches underwent an inclusion review process. Both primary reviewers (JF and JW) independently screened the titles and the abstracts of the citations identified by the electronic searches. Using the screening criteria in Table 2, the reviewers made eligibility judgments on whether studies should be included or excluded. Disagreements between reviewers’ recommendations were resolved by retrieving the paper and reaching a consensus after re-review.

**Table 2:** Study eligibility criteria

**Criteria**	**Response options**
Relevant population?	Yes/No/Unclear
Is the aim clearly stated?	Yes/No
Relevant Intervention?	Yes/No/Unclear
Relevant outcome measures?	Yes/No/Unclear

All studies which met the criteria for inclusion were retrieved. Both reviewers independently read all of the retrieved studies and recommended which studies should be included in the review. Disagreements that arose were reconciled by discussion.

### Study quality assessment

We adopted the quality criteria developed by Buckley et al. (2009), which has been utilised in a previous systematic review of workplace based assessment incorporating qualitative, quantitative and mixed methods studies, [7] to assess the quality of quantitative and qualitative studies included [18]. These criteria consist of a series of 11 quality ‘indicators’ (Table 3) with higher quality studies considered to be those which meet a minimum of seven indicators [18].

**Table 3:** Assessment of study quality

**Quality Indicator**	**Detail**
**Research question**	Is the research question(s) or hypothesis clearly stated?
**Study subjects**	Is the subject group appropriate for the study being carried out (number, characteristics, selection, and homogeneity)?
‘**Data’ collection methods**	Are the methods used (qualitative or quantitative) reliable and valid for the research question and context?
**Completeness of ‘data’**	Have subjects dropped out? Is the attrition rate less than 50%? For questionnaire based studies, is the response rate acceptable (60% or above)?
**Control for confounding**	Have multiple factors/variables been removed or accounted for where possible?
**Analysis of results**	Are the statistical or other methods of results analysis used appropriate?
**Conclusion**	Is it clear that the data justify the conclusions drawn?
**Reproducibility**	Could the study be repeated by other researchers?
**Prospective**	Does the study look forwards in time (prospective) rather than backwards (retrospective)?
**Ethical issues**	Were all relevant ethical issues addressed?
**Triangulation:**	Were results supported by data from more than one source?

### Data extraction

Two reviewers (JF and JW) independently undertook data extraction of all studies using a pre-designed proforma. The list below outlines the data retrieved from each article.

Information retrieved from included papers

1. Aim/objectives of study

2. Type of study and Study design

3. Setting and population

a. Location

b. Specialty

c. Number of doctors included in study

4. Information regarding MSF instrument

a. MSF questionnaire

b. Format of feedback

5. Kirkpatrick outcomes measured/reported

6. Key findings

a. Changes identified

b. Influences on change

7. Conclusions

## Results

**Figure 1:**
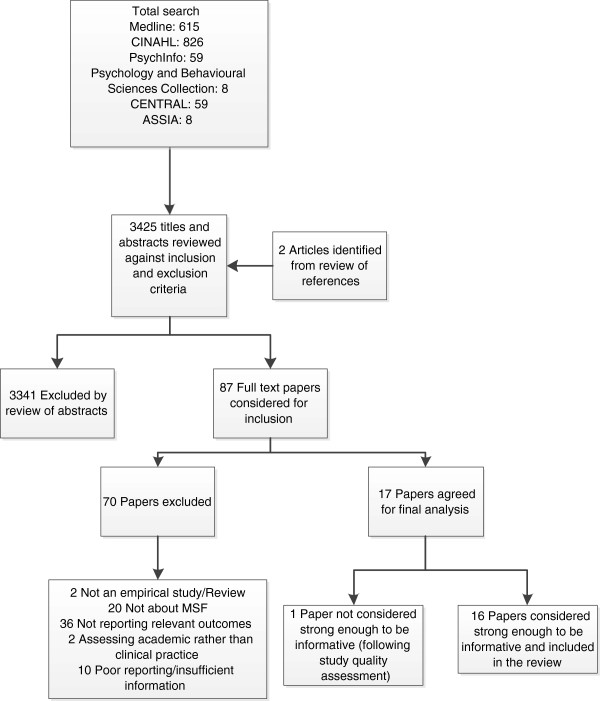
Review process.

The combined search of electronic databases yielded 3425 articles (Figure 1). After initial review, 87 were considered to be potentially relevant and the full article texts were obtained. Of these, 70 articles were then excluded as they did not meet the eligibility criteria (Table 3). The remaining 17 articles were assessed for study quality, with a further article being discounted, as it was deemed to be of insufficient quality. A total of 16 articles, therefore, met the inclusion criteria and were judged to be of sufficient quality to answer our study questions.

Included articles employed a range of study designs: RCT (n = 1); cross sectional survey questionnaires (n = 7); mixed methods (n = 2); and qualitative methods (n = 6). The studies focused on primary care physicians (n = 9), secondary care physicians (medical specialists (n = 3), surgeons (n = 1), paediatricians (n = 1)), foundation trainees (n = 1) and an unspecified physician group or setting (n = 1). The studies were conducted in Canada (n = 8), the UK (n = 3), the Netherlands (n = 3) and the USA (n = 2). A summary of study designs, populations and demographics is outlined in Table 4.

**Table 4:** Summary of characteristics of included studies

**First author and Date**	**Type of study**	**Participants**	**MSF tool**	**Feedback facilitated?**	**Feedback format**	**Influencing factors**	**Change identified**	**Kirkpatrick level**
Brinkman (2007) [19]	RCT	Paediatricians	Not specified	Yes: by a coach	Feedback report about baseline parent and nurse evaluations, and a tailored coaching session	Not discussed	Improved communication with patients & families. Improved demonstration of responsibility & accountability.	3b
Burford (2010) [20]	Quantitative: Cohort Study employing questionnaires	Foundation trainees	Mini Peer Assessment Tool (Mini-PAT), Team Assessment of Behaviour (TAB)	No	Confidential report	Highlighted the need for a facilitator Perceived validity of raters	Intention to change behaviour (no specific examples given)	3a
Fidler (1999) [21]	Quantitative Questionnaire survey & focus group	Family physicians	Physician Achievement Review (PAR)	No	Report	Negative mean feedback ratings	Improved communication with patients, better follow-up of patients. Improved written & verbal communication with health professionals	3a
Hall (1999) [22]	Quantitative Before & after study	Family Physicians	PAR	No	Confidential report	Identified need for facilitated feedback. Age of physician. Gap between peer rating and self rating	Improved communication with patients	3a
Lipner (2002) [23]	Mixed methods – focus groups & questionnaire	Physicians	Patient survey.	No	Confidential report	Not discussed	Intention to make changes by improving communication with patients (e.g. discuss treatment options more fully), improving communication with peers, and also participate in self-reflection	3a
			Peer Survey					
Lockyer (2003) [24]	Quantitative Before & after survey	Surgeons	Developed for study	No	Report	Age of physician. Gap between peer and self ratings	Making printed material available, maintaining medical records, managing stress & improving telephone access for patients.	3a
Overeem (2009) [25]	Qualitative – grounded theory interview study	Medical Specialists	PAR, American Board of Internal Medicine (AIM)	Yes: by a "mentor" or "coach"	Report	Facilitated feedback. Reflection on feedback. Self efficacy. Goal setting.	Performance improvement – e.g. improved communication with colleagues.	3a
Overeem (2010) [26]	Quantitative cross-sectional survey study	Medical Specialists	PAR, ABIM, Dutch Appraisal and Assessment Instrument (AAI)	Yes: a trained “facilitator”	feedback from colleagues, coworkers and/or patients summarized in a feedback report.	Facilitation Narrative comments	Intention to change professional performance & development of a personal development plan incorporating proposed changes.	3a
Overeem (2012) [27]	Quantitative observational and questionnaire evaluation study	Medical Specialists	Web-based MSF	Yes: by a "mentor"	Report consisting of the collation of MSF ratings from colleagues, coworkers and patients.	Perceived quality of mentoring. Negative scores.	Intention to change one or more aspects of professional performance.	3a
Owens (2010) [28]	Qualitative focus group and interview study	General Practitioners (trainees and doctors)	Not specified	No: Doctors. Yes: Trainees-a supervisor.	Report – however format of report varied.	Receiving several comments about the same behaviour	GPs improved communication with staff. Trainees improved their professional behaviour with staff & patients	3a
Sargeant (2003) [29]	Quantitative pilot study. Questionnaire evaluation survey	Family Physicians	PAR	No	Confidential report	Familiarity. Patient feedback Highlighted need for facilitated feedback	Intention to make or had made practice changes – mainly involving communication with patients (esp. written communication, phone communication, waiting times & accessibility)	3a
Sargeant (2005) [30]	Qualitative Focus groups	Family Physicians	PAR	No: contact provided if needed	Mailed confidential report	Unbiased yet informed raters. Agreeing with the feedback. Perceived usefulness of feedback. Negative influence – disagreeing with feedback	Examples of changes included improved communication with consultants & patients, improving information provided to patients following diagnostic tests	3a
Sargeant (2007) [31]	Qualitative Interviews	Family Physicians	PAR	No: contact provided if needed	Mailed confidential report	Familiarity with/credibility of rater. Facilitation. Emotional response. Negative feedback. Patient Feedback. Clear and specific feedback.	Improved communication with patients (e.g. providing fuller explanation) & co-workers. (e.g. improved written/verbal communication with pharmacists)	3a
Sargeant (2008) [32]	Qualitative Interviews	Family Physicians	PAR	No: contact provided if needed	Mailed confidential report	Negative feedback. Feedback inconsistent with their own self perceptions	Non-specific behaviour changes reported	3a
Sargeant (2009) [33]	Qualitative – grounded theory. Interview study	Family Physicians	PAR	No: contact provided if needed	Mailed confidential report	Reflection. Emotional response. Facilitation. Feedback inconsistent with their own self perceptions.	General behaviour changes	3a
Shepherd (2010) [34]	Mixed methods - questionnaire and interview study	General Practitioners	MSF developed for study	Yes: by appraiser	Confidential report – downloaded from a website.	Honesty on part of raters, appraisers and appraisees	Examples given included: improving systems used for communication, changing behaviour in interactions with colleagues, improving delegation	3a

### Impact of MSF on medical professionals’ professional practice

Of the 16 studies, only one, an RCT, identified a measured change in behaviour (Kirkpatrick level 3b) [19]. Alongside the MSF report, the intervention group also filled in a self assessment form and took part in a tailored coaching session to assist them in identifying their strengths and weaknesses and in setting specific behavioural goals. The 18 physicians who engaged in MSF demonstrated a significant improvement in a number of professional behaviours including communicating effectively with the patient and family (35%; 95% confidence interval, 11.0%-58.0%), timeliness of completing tasks (30%; 95% confidence interval, 7.9%-53.0%), and demonstrating responsibility and accountability (26%; 95% confidence interval, 2.9%-49.0%) compared to a control group of a further 18 who did not take part in the MSF. However, it is unclear whether this improvement would have occurred without the tailored coaching session. Furthermore, the wide confidence intervals reported in this study mean that these results need to be interpreted with caution and further research is therefore needed, to confirm the findings.

Three studies did not specify the types of changes made [20,32,33]. Of those that did, the most commonly reported change in behaviour related to improvement in communication, either with colleagues or patients [19,21,22,25,28,29,31,34]. Other changes included better follow-up of patients [21], maintaining medical records, [24] improving information provided to patients [24,30] and managing stress [24].

In two studies intention to change rather than actual change was reported and there was evidence that participants were more likely to contemplate and intend to change their behaviour in some way rather than actually implementing changes [23,29]. For example, one study of 356 participants found that 42% of the physicians expressed an intention to make changes in their communication with patients and 28% intended to change communication strategies with peers in response to the feedback [23]. However, the study did not explore whether these intended changes resulted in actual change. Also, as the authors acknowledge, it is unclear whether any changes made actually led to improvements in performance.

### Main factors of MSF that influence its impact on medical professionals’ professional practice

#### Source of the feedback

The influence of the raters on the participants’ acceptance and use of the feedback was highlighted by three of the studies [29-31]. A focus group study identified that feedback was only perceived as useful when physicians considered the raters (colleagues or co-workers) to be familiar with their work and had experience of either working with them or observing their practice [30]. This familiarity was related to how useful they perceived the feedback, namely if they felt that the rater was credible (was familiar with their work) the participants were more likely to perceive the feedback as useful and were more likely to consider changing their behaviour as a result [30]. Burford et al. (2010) [20] also highlighted that acceptance and use of feedback was influenced by whether the physician felt the rater had sufficient knowledge of their work. In their study participants reported that raters were mainly selected for the perceived value of their feedback [20]. Another study identified that the characteristics of the raters were found to be the main influence on physicians’ use of the feedback, especially honesty on the part of the raters [34]. This honesty was also highlighted as a factor by Burford et al. who identified that raters need to aim to be unbiased in their feedback [20]. Nonetheless, nearly half the raters in the study reported using indirect evidence (discussion with colleagues, absence of negative behaviour, inference from other observed behaviour) to assist in the development of their feedback, calling into question the usefulness of their feedback.

Feedback from patients was also found to have a positive influence on physicians’ acceptance and use of the feedback. Indeed, in two studies feedback from patients was shown to have a greater impact on behaviour change than feedback from colleagues [29,31]. In these two studies the main behavioural change made concerned communication with patients; this was probably due to the fact that patients directly observe the behaviour of this particular group (family doctors) to a greater extent than colleagues or co-workers. In addition, the MSF tool used - the Physician Achievement Review – included questionnaire items which provided a clear direction for physicians to change their communication behaviour [31].

#### Format of the feedback

In six of the studies feedback was facilitated, either by an appraiser, [34] mentor, [25,27] facilitator, [26] supervisor [28] or coach [19]. This facilitation was said to enable the acceptance of the feedback and the setting of achievable goals [26]. In one study, the quality of this facilitation was also key to the physicians’ acceptance and subsequent behavioural change [27]. In the other 11 studies the feedback was non-facilitated and in the form of a confidential report. While four of the studies identified that contact was provided if needed, they did not specify the format of this contact, how many physicians accessed this support and their reaction to this support [30-33]. In six studies, where the feedback was non-facilitated, a need for facilitated feedback was identified as it was felt that facilitated feedback would positively influence the acceptance of the feedback or the management of the emotional response associated with the feedback [20,22,26,29,32,33].

MSF reports can comprise numerical scores only or may incorporate narrative comments as well. There was evidence that participants preferred to receive written comments rather than just a numerical score [20,25]. While one study identified only a small, though significant, preference for free-text comments from raters, [20] another found that written comments from the raters were essential to the physicians satisfaction with and acceptance of the feedback [25].

#### Content of the feedback

Three studies identified that receiving negative comments influenced whether physicians contemplated or initiated changes [21,31,32]. For example, Fidler et al. found that physicians who had more negative ratings in their feedback report contemplated or initiated more behavioural changes than physicians who did not receive as many negative ratings [21]. Three of the studies found that when the physicians received positive feedback that confirmed good professional practice, they saw no need for change [31-33]. Receiving several comments from different sources about the same behaviour was also found to facilitate the use of the feedback received [28].

#### Response to the feedback

Two studies found that the process of reflecting on the MSF feedback was an important factor in its use [26,33]. Sargeant et al. found that reflection was integral to whether the feedback received was learned from and used to change professional practice [33]. The participants in this study highlighted that, for the feedback to be accepted and used, reflection needed to be facilitated, with the facilitator supporting and guiding the reflective process. However, it is unclear whether this reflection would have been as effective had it not been facilitated. This was mirrored by Overeem et al. (2009) who highlighted that the facilitator supported the reflective process ensuring that both strengths and weaknesses of the physician are focussed on [26].

The physicians’ emotional response to the feedback was also found to influence the acceptance and use of the feedback [30-33]. Physicians who received feedback that was negative and/or inconsistent with their self-perceptions, were more likely to respond with emotional distress [32,33]. In an interview study with 28 family physicians who had participated in MSF, Sargeant et al. (2008) found that while some eventually accepted the feedback and went on to initiate change, others did not and, rather than accept the feedback, questioned the feedback process [32].

In two of the studies, it was found that when the physicians disagreed with the feedback they questioned its credibility and asserted that they would not make any changes to their practice until they verified the responses by conducting their own independent reviews of their practice [30,31].

## Discussion

This systematic review synthesised the available evidence concerning the educational impact of MSF, defined by Overeem et al. (2012) as “the impact of MSF on change in practice”, [27] and its influence in changing behaviours and improving doctors’ performance. While a number of systematic reviews have looked at the effect of various types of workplace based assessment on the professional behaviours of medical doctors, to date no review has been carried out that focuses specifically on multisource feedback [7,8,11,35]. This review sought to address this gap.

Considering the importance now placed on workplace based assessment, in particular MSF, there are surprisingly few published articles exploring the impact of MSF on physician behaviour and the factors that influence the effectiveness of MSF. Research has tended to focus more on the validity and reliability of different tools and on the acceptability of MSF to those using this assessment tool, rather than on its educational impact on improving professional performance and behaviours where judged to be necessary.

One of the main factors identified that could enhance MSF acceptance and use was whether feedback is facilitated, by an appraiser, mentor or coach. Furthermore, in five of the studies where feedback was not facilitated, physicians identified a need for facilitated feedback [20,22,29,31,32]. It was felt that facilitation would assist doctors in accepting the feedback and in using it to change their behaviour [31,32].

It has been hypothesised that receiving negative or discrepant feedback (ratings from others that are lower than self-ratings) could be a catalyst for change, however evidence supporting this is limited [13]. In reality, it has been found that physicians who rated themselves higher than others are more likely to react negatively to the feedback and not change their behaviour [13] The influence of negative feedback, or feedback that was not consistent with their own self perceptions was also highlighted by six studies in this current review [21,22,26,30,31,33]. Specifically, Overeem et al. (2012) identified that the quality of the mentoring and the receipt of negative scores by colleagues were the main motivators for change for participating physicians [27]. The regression analysis undertaken found that the two variables, quality of mentoring and scores by colleagues (physicians who received lower ratings from their medical colleagues reported more change in practice) accounted for 34% of the variance [27]. While the interaction between these two variables is unclear in this study, two studies highlighted that the role of the facilitator is key to the physicians’ acceptance of the feedback [31,33]. The facilitator was said to encourage reflection and subsequent acceptance of the feedback which in turn led to a change in behaviour.

The findings of this review are similar to other reviews of MSF in non-healthcare settings. For example Smither et al. (2005) carried out a meta-analysis of 24 longitudinal studies of MSF in non-healthcare organisational settings, focussing on how managers use the MSF feedback over time [36]. The authors concluded that following participation in MSF, eight factors were identified as playing a part in influencing the extent of the change in behaviour and performance improvement [36]. These factors are:

Characteristics of the feedback, initial reactions to feedback, personality, feedback orientation, perceived need for change, beliefs about change, goal setting, and taking action [36].

As with this current review, if the recipients reacted negatively and rejected the feedback they were unlikely to use it to change their behaviour. However, unlike this current review, Smither (2005) did not look at the content or format of the feedback. Bracken and Rose (2011) carried out a review of the literature focussing on the factors of the MSF process that facilitates behaviour change, and like this current review, identified that the credibility of the rater was an important factor [37].

### Strengths and limitations

Strengths of the study were that a recognised systematic review process was followed [38] and papers meeting the study criteria were appraised by two researchers. Data extraction using a pre-designed proforma was independently undertaken by both researchers ensuring that no relevant data were overlooked. Applying Buckley et al’s [18] quality criteria ensured that studies of very poor quality were eliminated. To evaluate outcomes, Barr’s (2000) adaptation of Kirkpatrick’s four level evaluation model [17] was applied to all study findings; this ensured that only the outcomes relevant to the study were included.

This study has some limitations. Firstly, while the literature search was extensive and covered a number of databases, the articles retrieved were restricted to English-language publications only, and therefore publication bias cannot be ruled out. Secondly, methodological quality varied extensively between the articles and most of the studies were conducted on small volunteer-based samples. Also, as this review only focussed on published empirical studies some relevant unpublished studies may have been missed. Lastly, two studies included in this review have been reported in multiple articles. The articles by Fidler et al. (1999) [21] and Hall et al. (1999) [22] report on different aspects of the same study and the five articles reported by Sargeant et al. (2003, 2005, 2007, 2008, 2009) [29-33] all relate to the same sample population, from the pilot study reported by Sargeant et al. (2003) [29]. This could lead to over-reporting of the findings [39].

Whatever review method is employed, the resultant synthesis is only equivalent to the primary data included in the synthesis and the studies identified in this review had a number of limitations. Firstly, 15 of the 16 studies were descriptive and therefore the strength of the findings may be limited by the uncontrolled nature of the studies. However, it is asserted that, given the methodological difficulties of evaluating health professionals’ performance and the educational impact of interventions, descriptive and observational studies can still provide valuable information [7]. In fact, in this review the strongest evidence for improved performance following the MSF intervention and for the influencing factors for the effectiveness of the MSF intervention was provided by a focus group study, [27] and interview studies [31,33]. However, it should be noted that all three articles are from the same sample population and therefore publication bias may exist.

Fifteen of the 16 studies described self-reported rather than measured changes in behaviour. While self-reported measures of change in behaviour are highlighted as being quicker and easier than measuring actual behaviours, it is argued that healthcare systems are not concerned with changing health professionals self-reported behaviour, rather they want to change actual behaviour in the belief that this should lead to improved patient care [40]. Both Eccles et al. (2006) [40] and Hrisos et al. (2009) [41] purport that while self-reported change in behaviour can be a useful proxy for actual behaviour the extent to which these measures accurately reflect actual behaviour is unclear.

Some of the studies reported intention to change rather than actual change in behaviour [20,23,26,27]. A number of health psychology theories, such as the theory of planned behaviour [42-44] and protection motivation theory [45-47] assume that intentions (a person’s motivation or decision to engage in a course of action [47]) cause behaviour. However, as studies focussing on the intention-behaviour relationship have generally been correlational rather than causational this causality is still to be determined [48]. While Webb and Sheeran (2006) cite a number of meta-analyses that illustrate a relationship between intention and behaviour they highlight that this relationship could be in the reverse direction (that behaviour caused the intention) [48]. Therefore, it cannot be conclusively determined whether intention actually leads to behaviour change as a result of an MSF intervention.

### Implications for practice

An MSF tool which is designed to incorporate feedback from ‘credible’ raters (i.e. people who know the physician’s practice well, including patients) and which includes narrative comments as well as numerical scores is desirable. Furthermore, it seems to be helpful if the feedback is facilitated as this can influence the physician’s response to the feedback, how they deal with any negative comments and can promote reflection, all of which can impact on how the physician accepts and uses the feedback. Therefore when implementing an MSF intervention, particularly on an organisational or national basis, these factors need to be considered in order to optimise the effectiveness of the process.

### Implications for research

Considering the emphasis now placed on MSF as a method of formative performance assessment, there are surprisingly few published articles exploring these areas, and the strength of the study findings is questionable. While the studies identified in this review did examine both the behaviours influenced and the influencing factors, further research needs to be carried out to identify how the feedback influences behaviour, the strength of the influencing factors, and what impact behaviour change has on the quality and safety of patient care. In addition, only one study [15] looked at the change in behaviour over time, and in that study the follow-up was only five months. Therefore, it is not known whether the changes made are sustained over a longer period of time. Furthermore, while feedback is increasingly being used with health professionals other than primary and secondary care physicians, this review identified no eligible published studies involving these clinical groups which may be a cause for concern if MSF is also to be implemented as a performance measure by these professions.

The positive influence of a facilitator in the MSF process was highlighted in this review. However further research is clearly necessary to uncover the role and influence of the facilitator in the feedback process, particularly given the resource and logistical implications of providing such facilitation at scale as part of a national MSF system. Furthermore, research is also required to determine whether facilitation on its own is the key influence underpinning the impact of MSF, or in combination with the other factors identified as important (such as the source and format of the feedback).

## Conclusions

This review has identified that participation in MSF by medical doctors can lead to improved performance, although the volume and quality of supporting evidence is limited. Several factors were found to influence the extent to which professional performance is improved, which are summarised below.

### Key findings

Source of the feedback:

• The raters need to be perceived as credible and familiar with the work of the physician [29-31,34]

• Feedback from patients can increase acceptance of feedback and encourage positive change [29]

How the feedback is delivered:

• Facilitated feedback is desirable [20,22,25,26,29,31,33]

• Narrative comments from raters are valuable [26]

Content of the feedback:

• Negative comments may stimulate behavioural change, except where comments are inconsistent with physicians’ own perceptions [21,27,32,33]

• Receiving a number of comments about the same behaviour encourages acceptance and change [28]

• A gap between a peer rating and how the physician rates themselves encourages acceptance and change [22,24]

• Response to feedback – having time to reflect on the feedback was seen as an important facilitator for behavioural change [25,31,33]

## Competing interests

The authors declare that they have no competing interests.

## Authors’ contributions

JF conceived of and designed the study, carried out the database searches, study selection, data extraction and interpretation and drafted the manuscript. JW acted as a second reviewer, participating in the study selection and data extraction and in the drafting and revising of the manuscript. PB participated in the drafting of and revising of the manuscript. All authors read and approved the final manuscript.

## Pre-publication history

The pre-publication history for this paper can be accessed here:

http://www.biomedcentral.com/1472-6920/14/76/prepub

## References

[B1] Gopee N Self assessment and the concept of the lifelong learning nurse Brit J Nurs 2000 9 724 729 11235266 10.12968/bjon.2000.9.11.6264

[B2] Epstein RM Hundert EM Defining and assessing professional competence JAMA 2002 287 226 235 10.1001/jama.287.2.226 11779266

[B3] Epstein RM Assessment in medical education New Engl J Med 2007 356 387 396 10.1056/NEJMra054784 17251535

[B4] Hayes OW Reisdorff EJ Walker GL Carlson DJ Reinoehl B Using standardized oral examinations to evaluate general competencies Acad Emerg Med 2002 9 1334 1337 10.1111/j.1553-2712.2002.tb01597.x 12414491

[B5] Wilkinson TJ Wade WB Knock LD A blueprint to assess professionalism: results of a systematic review Acad Med 2009 84 551 558 10.1097/ACM.0b013e31819fbaa2 19704185

[B6] Norcini J Burch V Workplace-based assessment as an educational tool: AMEE Guide No. 31 Med Teach 2007 29 855 871 10.1080/01421590701775453 18158655

[B7] Miller A Archer J Impact of workplace based assessment on doctors’ education and performance: a systematic review BMJ 2007 341 710 715 10.1136/bmj.c5064 PMC2945627 20870696

[B8] Overeem K Faber MJ Arah OA Elwyn G Lombarts KM Wollersheim HC Grol RP Doctor performance assessment in daily practise: does it help doctors or not? A systematic review Med Educ 2007 41 1039 1049 10.1111/j.1365-2923.2007.02897.x 17973764

[B9] van der Vleuten CPM Schuwirth LWT Scheele MD Driessen EW Hodges B The assessment of professional competence: building blocks for theory development Best Pract Res Cl Ob 2010 24 703 719 10.1016/j.bpobgyn.2010.04.001 20510653

[B10] van der Vleuten CPM Norman GR De Graaf E Pitfalls in the pursuit of objectivity: issues of reliability Med Educ 1991 25 110 118 10.1111/j.1365-2923.1991.tb00036.x 2023552

[B11] General Medical Council Supporting information for appraisal and revalidation 2012 London: General Medical Council

[B12] Alexander DM How do 360 degree performance reviews affect employee attitudes, effectiveness and performance? University of Rhode Island, Schmidt Labor Research Center Seminar Research Series [online] 2006 1 12 [http://www.uri.edu/research/lrc/research/papers/Alexander_360.pdf]

[B13] Brett J Atwater L 360 degree feedback: Accuracy, reactions and perceptions of usefulness J Appl Psychol 2001 86 930 942 11596809 10.1037/0021-9010.86.5.930

[B14] Jamtvedt G Young JM Kristoffersen DT O'Brien MA Oxman AD Audit and feedback: effects on professional practice and health care outcomes Cochrane Db Syst Rev 2006 2 Art. No.: CD000259 10.1002/14651858.CD000259.pub2 16625533

[B15] Violato C Lockyer JM Fidler H Changes in performance: a 5‒year longitudinal study of participants in a multi‒source feedback programme Med Educ 2008 42 1007 1013 10.1111/j.1365-2923.2008.03127.x 18823520

[B16] Liberati A Altman DG Tetzlaff J Mulrow C Gotzsche PC Ioannidis JP Clarke M Devereaux PJ Kleijnen J Moher D The PRISMA statement for reporting systematic reviews and meta-analyses of studies that evaluate health care interventions: explanation and elaboration PLoS Med 2009 6 e1000100 10.1371/journal.pmed.1000100 19621070 PMC2707010

[B17] Barr H Freeth D Hammick M Koppel I Reeves S Evaluations of Interprofessional Education: A United Kingdom Review of Health and Social Care 2000 London: CAIPE

[B18] Buckley S Coleman J Davison I Khan KS Zamora J Malick S Morely D Pollard D Ashcroft T Popovic C Sayers J The educational effects of portfolios on undergraduate student learning: a Best Evidence Medical Education (BEME) systematic review. BEME Guide No. 11 Med Teach 2009 31 282 298 10.1080/01421590902889897 19404891

[B19] Brinkman WB Geraghty SR Lanphear BP Khoury JC Gonzalez Del Rey JA De Witt TG Britto MT Effect of multisource feedback on resident communication skills and professionalism: a randomized controlled trial Arch Pediat Adol Med 2007 161 44 49 10.1001/archpedi.161.1.44 17199066

[B20] Burford B Illing J Kergon C Morrow G Livingston M User perceptions of multi-source feedback tools for junior doctors Med Educ 2010 44 165 176 10.1111/j.1365-2923.2009.03565.x 20059677

[B21] Fidler H Lockyer JM Toews J Violato C Changing physicians' practices: the effect of individual feedback Acad Med 1999 74 702 10.1097/00001888-199906000-00019 10386101

[B22] Hall W Violato C Lewkonia R Lockyer J Fidler H Toews J Jennett P Donoff M Moores D Assessment of physician performance in Alberta: the physician achievement review Can Med Assoc J 1999 161 52 57 10420867 PMC1232653

[B23] Lipner RS Blank LL Leas BF Fortna GS The value of patient and peer ratings in recertification Acad Med 2002 77 S64 10.1097/00001888-200210001-00021 12377708

[B24] Lockyer J Violato C Fidler H Likelihood of change: a study assessing surgeon use of multisource feedback data Teach Learn Med 2003 15 168 174 10.1207/S15328015TLM1503_04 12855387

[B25] Overeem K Lombarts MJMH Arah OA Klazinga NS Grol RP Wollersheim HC Three methods of multi-source feedback compared: a plea for narrative comments and coworkers' perspectives Med Teach 2010 32 141 147 10.3109/01421590903144128 20163230

[B26] Overeem K Wollersheim H Driessen E Lombarts K Van De Ven G Grol R Arah O Doctors’ perceptions of why 360-degree feedback does (not) work: a qualitative study Med Educ 2009 43 874 882 10.1111/j.1365-2923.2009.03439.x 19709012

[B27] Overeem K Wollersheimh HC Arah OA Cruijsberg JK Grol RP Lombarts KM Factors predicting doctors’ reporting of performance change in response to multisource feedback BMC Med Educ 2012 12 52 10.1186/1472-6920-12-52 22781214 PMC3422186

[B28] Owens J Is multi-source feedback (MSF) seen as a useful educational tool in primary care? A qualitative study Educ Prim Care 2010 21 180 20515546 10.1080/14739879.2010.11493903

[B29] Sargeant JM Mann KV Ferrier SN Langille DB Muirhead PD Hayes VM Sinclair DE Responses of rural family physicians and their colleague and coworker raters to a multi-source feedback process: a pilot study Acad Med 2003 78 S42 10.1097/00001888-200310001-00014 14557092

[B30] Sargeant J Mann K Ferrier S Exploring family physicians' reactions to multisource feedback: perceptions of credibility and usefulness Med Educ 2005 39 497 504 10.1111/j.1365-2929.2005.02124.x 15842684

[B31] Sargeant J Mann K Sinclair D Van der Vleuten C Metsemakers J Challenges in multisource feedback: intended and unintended outcomes Med Educ 2007 41 583 591 10.1111/j.1365-2923.2007.02769.x 17518839

[B32] Sargeant J Mann K Sinclair D Van der Vleuten C Metsemakers J Understanding the influence of emotions and reflection upon multi-source feedback acceptance and use Adv Health Sci Educ Theory Pract 2008 13 275 288 10.1007/s10459-006-9039-x 17091339

[B33] Sargeant JM Mann KV van der Vleuten CP Metsemakers JF Reflection: a link between receiving and using assessment feedback Adv Health Sci Educ Theory Pract 2009 14 399 410 10.1007/s10459-008-9124-4 18528777

[B34] Shepherd A Lough M What is a good general practitioner (GP)? The development and evaluation of a multi-source feedback instrument for GP appraisal Educ Prim Care 2010 21 149 164 20515544 10.1080/14739879.2010.11493901

[B35] Veloski J Systematic revew of the literature on assessment, feedback and physicians’ clinical performance: BEME Guide No. 7 Med Teach 2006 28 117 128 10.1080/01421590600622665 16707292

[B36] Smither JW London M Reilly RR Does performance improve following multisource feedback? A theoretical model, meta-analysis and review of empirical findings Pers Psychol 2005 58 33 66 10.1111/j.1744-6570.2005.514_1.x

[B37] Bracken DW Rose DS When does 360-degree feedback create behavior change? And how would we know it when it does? J Bus Psychol 2011 26 183 192 10.1007/s10869-011-9218-5

[B38] Centre for Reviews and Dissemination Systematic Reviews CRD’s Guidance for Undertaking Reviews in Health Care 2008 Heslington: CRD, University of York

[B39] Von Elm E Poglia G Walder B Tramer MR Different patterns of duplicate publication JAMA 2004 291 974 980 10.1001/jama.291.8.974 14982913

[B40] Eccles MP Hrisos S Francis J Kaner EF Dickinson HO Beyer F Johnston M Do self-reported intentions predict clinicians' behaviour: a systematic review Implement Science 2004 1 28 10.1186/1748-5908-1-28 PMC1664582 17118180

[B41] Hrisos S Eccles MP Francis JJ Dickinson HO Kaner EF Beyer F Johnston M Are there valid proxy measures of clinical behaviour? A systematic review Implement Science 2009 4 37 10.1186/1748-5908-4-37 PMC2713194 19575790

[B42] Ajzen I Kuhl J, Beckmann J From intentions to actions: A theory of planned behavior Action-control: From cognition to behavior 1985 Heidelberg: Springer 11 39

[B43] Ajzen I Madden TJ Prediction of goal-directed behavior: attitudes, intentions, and perceived behavioral control J Exp Soc Psychol 1986 22 453 474 10.1016/0022-1031(86)90045-4

[B44] Ajzen I Attitudes, personality and behavior 1988 Chicago: Dorsey Press

[B45] Rogers RW A protection motivation theory of fear appeals and attitude change J Psychol 1975 91 93 114 10.1080/00223980.1975.9915803 28136248

[B46] Rogers RW Cacioppo BL BL, Petty LL Cognitive and physiological processes in fear appeals and attitude change: A revised theory of protection motivation Social psychophysiology: A source book 1983 London: Guildford Press 153 176

[B47] Rogers RW Attitude change and information integration in fear appeals Psychol Rep 1985 56 179 182 10.2466/pr0.1985.56.1.179

[B48] Webb TL Sheeran P Does changing behavioral intentions engender behavior change? A meta-analysis of the experimental evidence Psychol Bull 2006 132 249 268 16536643 10.1037/0033-2909.132.2.249

